# Comparison of three fluids for calibration of the new Periotron® 8010

**DOI:** 10.4317/medoral.25917

**Published:** 2023-04-26

**Authors:** Meylin Fernández-Reyes, Cecilia Fabiana Márquez-Arrico, Francisco Javier Silvestre, Laura Perea-Galera, Javier Silvestre-Rangil, Milagros Rocha

**Affiliations:** 1 Department of Endocrinology and Nutrition, University Hospital Doctor Peset-FISABIO, Valencia, Spain; 2 Department of Stomatology, University Hospital Doctor Peset-FISABIO, Valencia, Spain; 3 Department of Stomatology, University of Valencia, Valencia, Spain; 4 CIBER CB06/04/0071 Research Group, CIBER Hepatic and Digestive Diseases, University of Valencia, Valencia, Spain

## Abstract

**Background:**

The aim of the present study was to calibrate the Periotron® model 8010 with volumes of three different fluids (distilled water, serum, and saliva) and to identify which of the three is the most reliable, feasible, and reproducible for routine calibration.

**Material and Methods:**

A total of 450 samples of Periopaper® were divided into three groups (150 each per group): distilled water, serum matrix and saliva. A calibration curve was run with 0.25, 0.50, 0.75, 1.00 and 1.25 µl of each of the fluids, and the results were determined in Periotron units (PU). Statistical analysis was performed with one-way ANOVA followed by Bonferroni’s post hoc test and a linear equation.

**Results:**

Distilled water presented the lowest levels of PU at all volumes, while serum showed the highest levels at high volumes. Linear regression equations rendered similar slopes for saliva and distilled water, while serum was statistically different. Saliva presented a reproduction percentage of 99.7%, which indicated better accuracy and precision than serum and distilled water.

**Conclusions:**

Saliva is more reliable and accurate than water or serum for the purpose of calibration of the Periotron® model 8010, though it shares drawbacks with serum. Distilled water is more easily available and does not require any additional procedure, in addition to producing a similar slope to saliva and a smaller deviation from the media than serum.

** Key words:**Gingival crevicular fluid, periodontics, calibration, periodontal disease.

## Introduction

Periodontal disease (PD) is a complex and multifactorial condition that directly affects the supporting structure of the teeth. It involves inflammatory mechanisms produced by the accumulation of bacterial plaque on dental surfaces. In this context, local, environmental and genetic factors can influence the interrelation between the subgingival biofilm and the host immune response (1,2). The most widely used method to determine periodontal pocket disease is a millimeter periodontal probe. However, probing does not establish the exact status of the periodontal pocket, which represents a considerable limitation (3). These limitations have motivated the use of alternative techniques, such as radiographic images and determination of gingival crevicular fluid (GCF) volumes, and the measurement of inflammatory markers, such as prostaglandins, protolithic or hydrolytic enzymes, and interleukins, as well as culture techniques and microscopy to detect bacteria (4,5).

Over the last few decades, GCF has been studied with the aim of improving periodontal diagnosis and therapy. GCF is an exudate of varying composition found in small amounts in the gingival sulcus (6). The route of GCF diffusion is through the basement membrane and subsequently through the junctional epithelium into the sulcus. Due to the fact that blood is filtered through the junctional epithelium, GCF contains components of serum, inflammatory cells, connective tissue, epithelium, and microbial flora that inhabit the gingival margin or the sulcus/pocket. It includes cellular elements such as epithelial cells, leukocytes, bacteria, electrolytes, albumin, alpha-globulins, immunoglobulins (Ig) type IgG, IgM and IgA, complement proteins, interleukins, cytokines and proteolytic and hydrolytic enzymes of inflammatory cell origin (7-9). During the inflammatory process, the GCF flow increases and its composition changes in a way that the presence of inflammatory markers augments. This increased GCF flow contributes to the host defenses by flushing bacterial colonies and their metabolites away from the sulcus. These variations in the volume of the GCF are directly proportional to the inflammatory expression, and even to the degree of periodontal inflammation (9-12). Currently, pipettes and paper strips are used to collect and determine GCF volume. Quantification of GCF as part of the management of PD is based on the possibility of identifying the presence of various enzymes, inflammation markers, and host cell debris that indicate different levels of periodontitis (13-15).

A new model of the Periotron®, the Periotron® 8010, has recently become available on the market. It quantifies the volume of GCF and saliva collected on Periopaper® in periotron units (PU) by measuring the electrical capacitance of the wet paper strip. As a result of opposing charges on the electrodes, an electric field is created, generating polarity in the molecules that reduces the potential difference between the electrodes, thus increasing the capacitance (14). Periopaper® is also used to detect microbiological or biochemical changes in the GCF (3,15-18), as the measurement of GCF volume is crucial in order to diagnose the severity of PD. Calibrating studies have previously been conducted with older models of Periotron® to confirm their reliability and reproducibility, aspects that are essential to the efficacy of the procedure (9,18-23). However, no such studies have been carried out with Periotron® 8010. For these reasons, the aim of the present study was to calibrate Periotron® 8010 with widely used volumes of distilled water, serum, and saliva and to identify which of the three is the most reliable, feasible, and reproducible for routine calibration. Furthermore, we evaluated and compared the calibration curves produced in each case.

## Material and Methods

The study was conducted at University Hospital Doctor Peset-FISABIO in Valencia (Spain) between November 2021 and March 2022. The total sample size [450] was divided into three groups according to calibration fluid; that is, 150 samples of distilled water (Quimipur®, Quimipur S.L.U, Madrid, Spain), 150 samples of serum matrix (Milliplex®, Millipore Corporation, Billerica, MA, USA) and 150 samples of saliva from a single healthy volunteer. Saliva was stimulated by chewing paraffin gum and was collected in a sterilized tube (15 ml) by means of 5-minute expectoration. The samples were centrifuged at 3,000g (Ortoalresa®, Alvarez Redondo.S.A, Madrid, Spain) at room temperature for 15 minutes to remove solid residues and the supernatant was placed in a sterile tube.

The Periotron® Model 8010, 450 sterile Periopaper® (PerioPaper®, Oraflow Inc., New York, USA) and a micropipette (Pipetman 0.2- 2 µl, Gilson Incorporated, Middleton, USA) were employed throughout the experiment, and data were collected following calibration by a single investigator on each day of the experiment (90 days).

- Periotron Protocol

The apparatus was always used in the same position and in dry conditions.

Autocalibration is performed when the Periotron® is "zeroed" by inserting a sterile or unused Periopaper® into the system. The manufacturer recommends allowing the Periotron® to warm up for 10 minutes before use. The calibration curve was run with 0.25, 0.50, 0.75, 1.00 and 1.25 µl of the corresponding calibration fluid and the results were determined in PU.

Periopaper® strips were placed immediately on the sensors in order to minimize errors caused by evaporation of the sample. It was also verified that the orange area was not in contact with the sensors and that the Periopaper® was placed on the middle of the sensors.

- Sample size calculation

The study was designed in order to detect minimum expected difference in Periotron units (PU) ≥5 among the three groups in terms of the primary efficacy criterion, assuming a common standard deviation of ≥5 units with a power of 90% and an α risk of 0.05. Under these premises, at least 28 samples per group were considered.

- Statistical analyses

Statistical analysis was performed with SPSS 24.0 software (IBMCorp, NY, USA). Quantitative variables were expressed as mean and standard deviation (SD).

One-way ANOVA followed by a Bonferroni post hoc test was used to compare precise volumes of the three different solutions (0.25, 0.50, 0.75, 1.00, 1.25 µl). Additionally, the relationship between the explanatory variable (volume) and a response variable (periotron units) was evaluated by fitting a linear equation. Slopes of different linear regression equations were compared by one-way ANOVA followed by a post hoc test in order to determine similarities (or the lack of them) among the three different equations.

A confidence interval of 95% was determined for all the tests, and differences were considered statistically significant when *p* < 0.05.

## Results

Differences between volumes of water, serum, and saliva were statistically different according to the means PU for each solution (Table 1). Of the three solutions, distilled water had the lowest levels of PU at all volumes, while serum presented the highest levels at high volumes (0.75, 1, 1.25µl).

**Figure 1:**
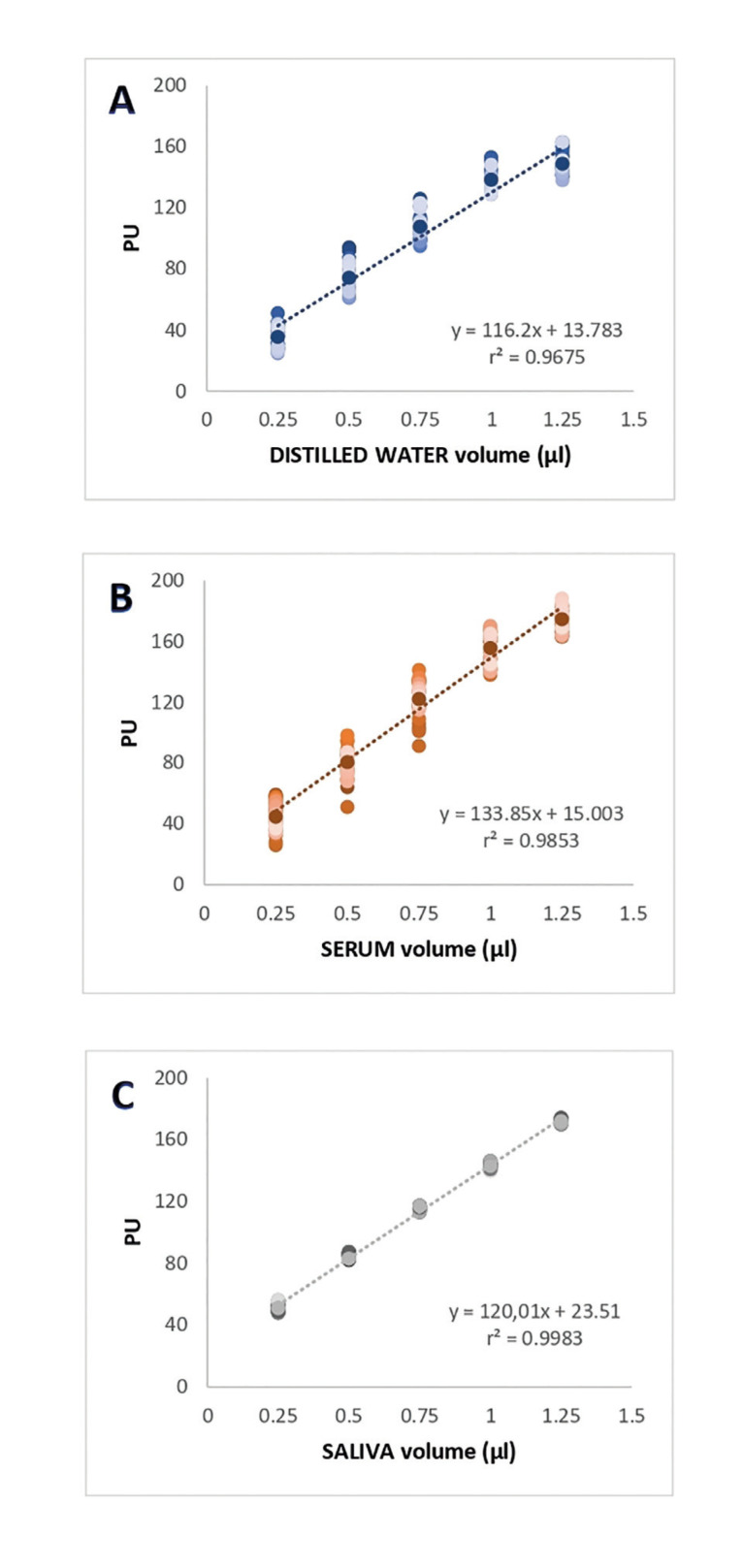
Plots of Periotron units (PU) scores with respect to number of replications of distilled water (A), serum (B) and saliva (C).

In terms of dispersion of the data with respect to the mean, Table 1 and Fig. 1 illustrate that saliva showed the lowest SD while serum showed the highest.

**Figure 2:**
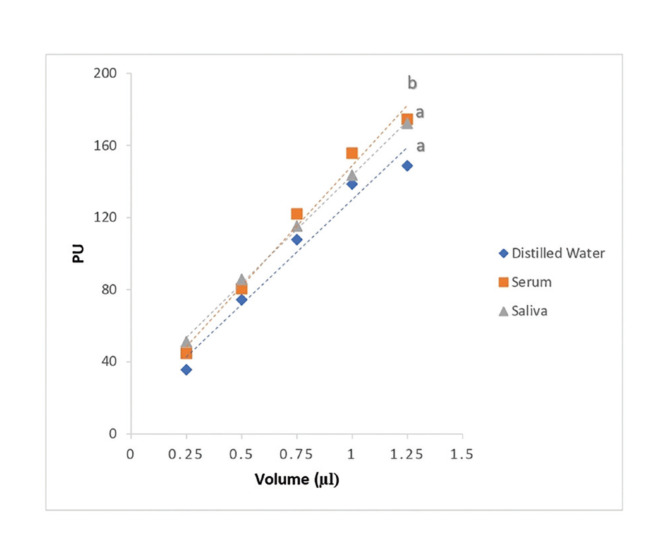
Plots of Periotron units (PU) scores with respect to slopes of linear regression produced by serum, saliva and distilled water.

When linear regression equations were performed, the slopes of saliva and distilled water proved to be similar, while serum was statistically different from saliva and distilled water (Fig. 1, Fig. 2).

**Table 1:**
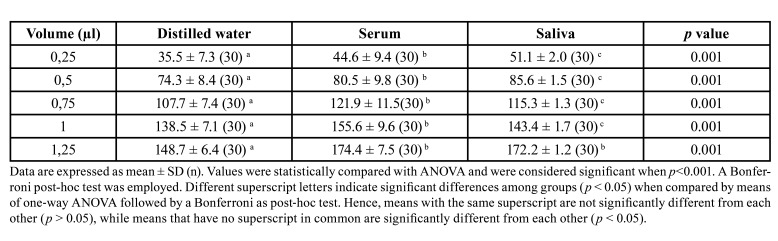
Determinations of Periotron® units (PU) in different volumes of distilled water, serum and saliva.

When we explored r2, which is a goodness-of-fit measure for linear regression models, the highest values were observed for saliva, followed by serum and distilled water, respectively (Fig. 1). Therefore, as a whole, our results suggest that serum is the least appropriate fluid to carry out the standard curve of Periotron® due to a high dispersion of values and higher and significantly different slope with respect to distilled water and saliva. Meanwhile, saliva and distilled water presented similar slopes, although PUs were significantly lower and showed greater dispersion in the case of distilled water.

The correlation coefficient between the calibration volumes of distilled water and PU was strong, with a prediction percentage of 93.9% (Fig. 1). The calibration volumes obtained with serum were similar to those of saliva, with an r2 of 0.985 (Fig. 2). The graph also presents plots of the PU scores for number of replications tested and precision in the case of serum. Saliva presented a reproduction percentage of 99.7%, and accuracy and precision were better in comparison to serum and distilled water. (Fig. 1).

As can be seen in Fig. 2, the linear regression analysis revealed that the slopes of the line were similar for saliva and distilled water, but different for serum.

## Discussion

In recent years, Periotron® has become a vital tool both in diagnosing and monitoring periodontal disease, since GCF volume is directly proportional to the degree of periodontal inflammation (11,13). However, a precise calibration is essential in order to obtain consistent results with the device. Some studies have investigated the differences between calibration substances and their influence on PU results (11,13,18,21-24), though none of them has been carried out with the device’s latest version, the Periotron® 8010.

The manufacturer recommends using serum, water or saliva interchangeably when calibrating the instrument. That said, distilled water and serum were used in most of the studies published to date (11,13,18,21-24). In the present study we have included saliva due to its similarity to GCF in terms of composition and viscosity (8-10).

The data we have obtained confirm that the substances that best fit a straight line are saliva and serum, while distilled water is the one that fits the least. On the other hand, the slopes of the regression line were similar for distilled water and saliva and different for serum. In this sense, it should be taken into account that distilled water in higher volumes tends to produce a saturation curve. According to our results, saliva was the most consistent substance when calibrating the Periotron® 8010, since it presented greater precision and accuracy than either distilled water or serum. However, saliva has certain drawbacks, as it requires a more laborious collection process and subsequent centrifugation to avoid contamination by leftover food. For this reason, and due to its easy accessibility, distilled water is generally used as a calibration standard.

In previous studies, it has been reported that water rendered higher PU readings than serum (22,24). Indeed, water has a very high electrical constant compared to proteins and ions, which means that values depend predominantly on the amount of water found in the serum. Strikingly, our PU readings were higher in serum than in distilled water, in contrast with that reported by previous studies (21-24), probably due to the different additives present in commercial serum matrixes.

Tözüm *et al*. also observed different readings for serum and distilled water, though they were significant only at high volumes (24). Nevertheless, as far as we know, no study has evaluated the reliability of calibration curves in saliva. Different solutions render different readouts due to their specific biochemical composition, which can include a high protein content, thus leading to greater variability (15). Additionally, other physical properties of the substance, such as viscosity, pH, evaporation rate, temperature and humidity, may have an influence on the reliability of calibration data (21). In order to minimize this variability, we carried out the measurements under controlled time intervals, temperature and humidity conditions.

The evaporation rate was also taken into consideration to reduce variability among our results. Previous studies have not reported clear differences in the evaporation rate of distilled water during the first 5-10 seconds, although evaporation has been shown to increase with transfer time, showing progressively greater evaporation at 30 and 60 seconds (24). For this reason, our sampling times did not exceed 10 seconds. Some studies have reported that room temperature and humidity can also modify electronic readouts (22,24); for example, roughly a 10% error range has been attributed to room temperature and humidity regardless of the calibration liquid employed (22). Generally, increased room temperatures generate higher readings and humidity can modulate this response, and other local circumstances can also cause readings to vary (24). In our study, room temperature and humidity were maintained constant under controlled conditions in order to obtain consistent results among the different calibration liquids.

In addition, based on the results of the present study, we can affirm that the placement of paper strips between the electrodes of the device is an important factor to be considered if volumetric distortions of electronic readings are to be minimized, as reported previously (19).

The principal strength of our study is that we are the first group to evaluate the use of different calibration fluids with Periotron® 8010, including saliva. Furthermore, all the experimental procedures were carried out by an experienced qualified operator.

To conclude, the Periotron® 8010 renders reproducible and reliable results with different calibration liquids; namely, distilled water, serum and saliva. Although saliva appears to be more reliable and accurate than water or serum, it shares several drawbacks with serum, as it requires collection from volunteers and centrifugation prior to use. On the other hand, distilled water is easy available and does not involve any additional procedure before the calibration curve can be performed. Moreover, it produces a similar slope to saliva and a lower deviation from the media than serum. Therefore, due to its feasibility, distilled water should generally be recommended as the primary calibration fluid for use with Periotron® 801 on a day-to-day basis.
